# Fusion primer and nested integrated PCR (*FPNI-PCR*): a new high-efficiency strategy for rapid chromosome walking or flanking sequence cloning

**DOI:** 10.1186/1472-6750-11-109

**Published:** 2011-11-17

**Authors:** Zhen Wang, Shafei Ye, Jingjing Li, Bo Zheng, Manzhu Bao, Guogui Ning

**Affiliations:** 1 Key laboratory of Horticultural Plant Biology, Ministry of Education, College of Horticulture and Forestry Sciences, Huazhong Agricultural University, Wuhan 430070, P. R. China

## Abstract

**Background:**

The advent of genomics-based technologies has revolutionized many fields of biological enquiry. However, chromosome walking or flanking sequence cloning is still a necessary and important procedure to determining gene structure. Such methods are used to identify T-DNA insertion sites and so are especially relevant for organisms where large T-DNA insertion libraries have been created, such as rice and *Arabidopsis*. The currently available methods for flanking sequence cloning, including the popular *TAIL-PCR *technique, are relatively laborious and slow.

**Results:**

Here, we report a simple and effective fusion primer and nested integrated PCR method (*FPNI-PCR*) for the identification and cloning of unknown genomic regions flanked known sequences. In brief, a set of universal primers was designed that consisted of various 15-16 base arbitrary degenerate oligonucleotides. These arbitrary degenerate primers were fused to the 3' end of an adaptor oligonucleotide which provided a known sequence without degenerate nucleotides, thereby forming the fusion primers (FPs). These fusion primers are employed in the first step of an integrated nested PCR strategy which defines the overall *FPNI-PCR *protocol. In order to demonstrate the efficacy of this novel strategy, we have successfully used it to isolate multiple genomic sequences namely, 21 orthologs of genes in various species of Rosaceace, 4 *MYB *genes of Rosa rugosa, 3 promoters of transcription factors of Petunia hybrida, and 4 flanking sequences of T-DNA insertion sites in transgenic tobacco lines and 6 specific genes from sequenced genome of rice and *Arabidopsis*.

**Conclusions:**

The successful amplification of target products through *FPNI-PCR *verified that this novel strategy is an effective, low cost and simple procedure. Furthermore, *FPNI-PCR *represents a more sensitive, rapid and accurate technique than the established *TAIL-PCR *and *hiTAIL-PCR *procedures.

## Background

The advent of the genomics era has had a massive impact on almost all fields of biological enquiry. However, it is still necessary to employ chromosome walking and flanking sequence cloning procedures in order to determine full gene structure. Such methods are also required to identify T-DNA insertion sites, and this is particularly relevant for species where large T-DNA insertion libraries exist, eg. rice and Arabidopsis [1-3]. Currently, a number of PCR-based methods are available for these purposes, including random PCR, inverse PCR, panhandle PCR, and cassette PCR. However, each of these methods has drawbacks when considered for wide use in the amplification of target regions. For example, inverse PCR is rarely used for chromosome walking due to problems with the availability of restriction sites in the unknown/known regions, and/or poor circularization of the template molecule [3,4]. Ligation-mediated PCR has proved to be too inefficient for routine use in flanking sequence identification, with non-specific amplified products accounting for the major proportion of the final PCR products [2,4,5].

In recent years, improved methodologies have been developed to address the problems outlined above [3-11]. The most successful of these involve PCR procedures using random primers, notably, the thermal asymmetric interlaced (*TAIL*)*-PCR *method. Such methods are relatively low cost and have the advantage of being relatively direct, i.e. there is no requirement for intermediate steps involving restriction enzyme digestion of the DNA template or ligation reactions [3-5]. Consequently, *TAIL-PCR *has been widely used, notably for the amplification of junction DNA at T-DNA insertion sites [1,12-14], the rapid isolation of promoter sequences [6], and the cloning of full length functional genes [9,15]. However, *TAIL-PCR *is a time consuming procedure that usually involves three rounds of amplification with each round consisting of multiple PCR cycles. The method often produces nonspecific amplification products due to the short arbitrary degenerate (AD) primers and low annealing temperatures, and may also achieve non-efficient amplification of target sequences [2,4]. Furthermore, conventional *TAIL-PCR *tends to produce small amplification products [5,16,17]. *TAIL-PCR *has subsequently been adapted, but the improved high-efficiency (*hi*) *TAIL-PCR *version still remains time consuming, and is expensive in terms of primer design and synthesis. The *hiTAIL-PCR *technique also poses a great challenge to the activity of DNA polymerase, and so is frequently associated with inefficient amplification results.

Recently, several alternative methods have emerged to perform chromosome walking or flanking sequence cloning. However, such methods still have the drawback that they are either restriction enzyme-dependent, Phi29 DNA polymerase-dependent, or require a series of template DNA purification steps [2,6,17]. Thus, these alternative methods are still laborious and do not display the wide application potential of conventional *TAIL-PCR*. Therefore, it is highly desirable that further novel methods are developed for rapid and efficient chromosome walking or flanking sequence cloning.

In order to acquire precise flanking fragments rapidly, we have developed a novel efficient method, namely fusion primer and nested integrated-PCR (*FPNI-PCR*). We believe that this method has the potential to have wide-spread application in genetics and genome walking studies, and is applicable throughout diverse organisms. In order to demonstrate the power of *FPNI-PCR*, we performed long PCR walking tests on various genomic targets namely, *FT*, *TFL1 *and *SOC1 *orthologs from the *Spiraea cantoniensis, Pyracantha fortuneana, Photinia serrulata, Fragaria ananassa, Rosa hybrida, Prunus mume *and *Prunus yedoensis *species of *Rosaceace*, 4 *MYB *genes of *Rosa rugosa*, 3 promoters from MADS-box transcription factors of *Petunia hybrida*, and 4 flanking sequences of T-DNA insertion sites in *Nicotiana*. In each case, successful amplification was achieved of the unknown genomic DNA fragments (> 100 kb sequences). In addition, comparison with genomic sequence databanks confirmed that *FPNI-PCR *successfully amplified various lengths of the flanking regions of 3 members of the *wuschel *gene family from *Arabidopsis*, also the flanking regions of the *Osft, Osmads1 *and *Ostuba1 *genes from rice, and an archived flanking sequence from the pCAMBIA2300 vector. Furthermore, a comparative analysis with conventional *TAIL-PCR *indicated that our novel *FPNI-PCR *method is more flexible, time saving and powerful than *TAIL-PCR *or *hiTAIL-PCR*. Thus, *FPNI-PCR *has high potential for application in identifying tagged sequences, or for genomic walking from known DNA regions toward unknown regions in organisms with a large genome.

## Results

### Principle of *FPNI-PCR*

**Figure 1:**
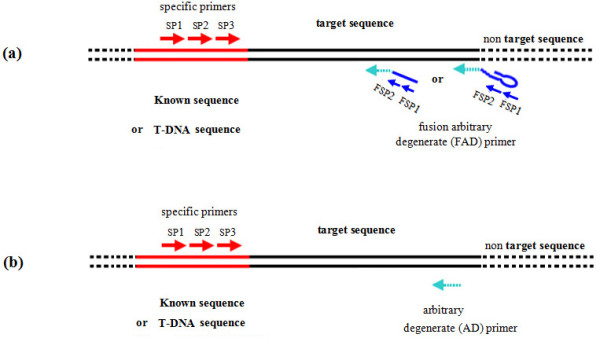
**Schematic outline of the differences in amplification of target and non-target sequences using *FPNI-PCR *(a) and *TAIL-PCR *(b)**. Black and dotted lines represent the target and non-target regions, respectively. Red lines represent the region of known genomic sequence (or known sequence of T-DNA or transposon elements integrated in the genomic DNA). Each red arrow denotes a specific primer designed from the known nucleotide sequence. A blue/green chimeric arrow denotes the FP primer used in the *FPNI-PCR *method. Each blue arrow denotes a specific primer designed from the known adaptor sequence.

**Figure 2:**
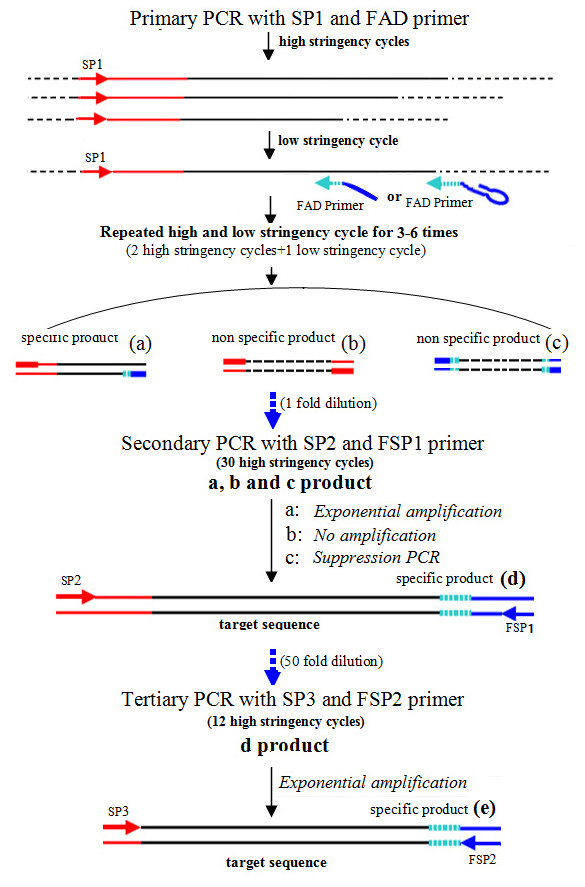
**A general (theoretical) scheme for *FPNI-PCR *(PCR based method for genomic walking or tagged flanking sequence cloning)**. In the first PCR step, single stranded copies of the target template are generated in the high stringency cycles, and double stranded products are produced in the low stringency cycle (in total, involving 3-5 repeated PCR cycles); in this primary step, amplification of the target products is likely to be accompanied with other, nonspecific, products. In the secondary and tertiary PCR steps (nested PCR), the target DNA is exponentially amplified by the gene specific and adaptor specific primers, while non-target genes are not amplified because there is no corresponding gene specific primer (and/or amplification was suppressed by the stem-loop structure of the DNA).

The basic principle of *FPNI-PCR *is outlined in Figure 1 and 2. The method centers on a series of primers encompassing sequence-specific primers (designed on regions of known DNA sequence), and fusion primers which contain an arbitrary degenerate (AD) section fused to a section of determined sequence (fusion primers). The fusion of a known adaptor of determined sequence to the 5'-end of an AD (or other short site-dependent primer) is the main characteristic of *FPNI-PCR *(Figure 1a), and it is this trait which differentiates *FPNI-PCR *from *TAIL-PCR *(Figure 1b). The *FPNI-PCR *protocol contains three key steps. In the first step, a large complex mixture of DNA reactions is prepared using 0.4 μL of a gene-specific primer (SP1) designed to the genomic region of known sequence, and 2.0 μL of a combination of nine fusion arbitrary degenerate primers (eg. FP1-9; Table 1); other designed primers for *FPNI-PCR *are presented in Additional files 1 Tables S1-S5. The details of cycling parameters and PCR conditions used in this study are listed in Table 2. This first step consists of 3-6 repeats of two high stringency cycles followed by a low stringency cycle. Theoretically, single stranded PCR products from the gene-specific primer are generated during the high stringency cycles, and double-stranded products utilizing the FP primers (FPs) are developed during the low stringency cycle. After 3-6 repeated cycles of this PCR regime, it is predicted that the intended target products are partially synthesized and are accompanied by other, nonspecific, products (Figure 2). In the second and third steps, nested PCR is conducted using 1 uL of target-specific primers (SP2/SP3, respectively) and FP-specific primers (FSP1/FSP2, respectively). These steps are high stringency PCR using high annealing temperatures so that target products are selectively amplified. Nonspecific products are not amplified in these steps, in part due to the nested approach with different primers. In addition, the large hairpin structure employed in some nonspecific products also contributes to prevent to further amplification (suppression PCR). Thus, the nonspecific products generated from the first PCR step in *FPNI-PCR *are not amplified in the second and third steps, and become substantially diluted in the final mix (Figure 2).

**Table 1:** FP primers (no hair pin structure) and universal primers used in *FPNI-PCR*.

Name	Primer sequence 5'-3'	Primer use
*FP1: *	GTAATACGACTCACTATAGGGCACGCGTGGT NTCGA STWTS GWGTT	1st PCR primer
*FP2:*	GTAATACGACTCACTATAGGGCACGCGTGGT NGTCG ASWGA NAWGAA	1st PCR primer
*FP3:*	GTAATACGACTCACTATAGGGCACGCGTGGT WGTGN AGWAN CANAGA	1st PCR primer
*FP4: *	GTAATACGACTCACTATAGGGCACGCGTGGT AGWGN AGWAN CAWAGG	1st PCR primer
*FP5: *	GTAATACGACTCACTATAGGGCACGCGTGGT NGTAW AASGT NTSCA A	1st PCR primer
*FP6:*	GTAATACGACTCACTATAGGGCACGCGTGGT NGACG ASWGA NAWGAC	1st PCR primer
*FP7: *	GTAATACGACTCACTATAGGGCACGCGTGGT NGACG ASWGA NAWGAA	1st PCR primer
*FP8: *	GTAATACGACTCACTATAGGGCACGCGTGGT GTNCG ASWCA NAWGTT	1st PCR primer
*FP9: *	GTAATACGACTCACTATAGGGCACGCGTGGT NCAGC TWSCT NTSCTT	1st PCR primer
*FSP1: *	GTAATACGACTCACTATAGGGC	2nd PCR primer
*FSP2:*	ACTATAGGGCACGCGTGGT	3rd PCR primer

**Table 2:** Cycling parameters and PCR conditions for *FPNI-PCR*.

PCR reaction	Cycle number	Thermal condition	Annotation
*First PCR*	1	95°C 90 s	*
	2	94°C 10 s; 62°C 30 s; 72°C 2 min	
	1	94°C 10 s; 25°C2 min; 0.2°C/s;72°C 2 min	
		94°C 10 s; 62°C 30 s; 72°C 2 min	
		94°C 10 s; 62°C 30 s; 72°C 2 min	
		94°C 10 s; 44°C 30 s; 72°C 2 min	
	1	72°C 5 min.	
*Second PCR*	1	95°C 90 s	**
	30	94°C 10 s; 62°C 30 s; 72°C 2 min	
	1	72°C 5 min.	
*Third PCR*	1	95°C 90 s	***
	12	94°C 10 s; 62°C 30 s; 72°C 2 min	
	1	72°C 5 min.	

Note: * = Few cycles of thermal asymmetric interlaced PCR: target product generation; incorporation of suppression PCR inverted repeat elements in partial non specific product; ** Standard high stringency PCR: Target Sequence exponential amplification; partial nonspecific products suppression PCR; *** = Standard Nested-PCR: completely eliminating high background or nonspecific amplification in the second PCR.

### Parameters in the design and optimization of *FPNI-PCR*

We compared two types of AD primers. In the basic form, i.e. type I primers, the AD primer was fused to the 3' end of an adaptor of known sequence (Table 1). Type II primers included a hairpin structure at the 5'end of the single-stranded adaptor (Additional files 1, Table S2). We compared the results from such type I and type II primers by testing them in combination with the same gene-specific (SP) primers and under the same cycling parameters. The final results of amplification of the target products indicated no obvious differences between the two primer types, i.e. they presented similar sizes of products on the agarose gels (data not shown). Thus, the hairpin structure at the 5'end of the type II single-stranded adaptors had no apparent impact on amplification of the target. Type III primers describe arbitrarily degenerative primers with 4-6 fixed nucleotides at the 3' end (Additional files 1, Table S3), and these primers were also successfully used to amplify the flanking regions of known fragments, with the various sizes of the product fragments relating to the given fusion primer.

**Figure 3:**
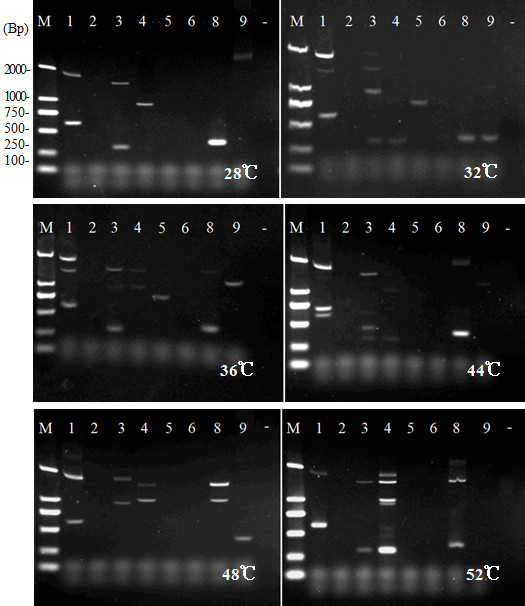
**The effect of annealing temperature during the low stringency PCR cycles of the primary PCR step**. Illustration of the effect of annealing temperature during the low stringency PCR cycles of the primary PCR step in *FT *ortholog cloning of *Fragaria ananassa *using *FPNI-PCR *(amplified products shown after the tertiary round of PCR). M: molecular marker; number in the lanes: the 1-9 FP primer; number in the bottom right corner: annealing temperature. -: control.

The primary round of PCR in the *FPNI-PCR *procedure includes an optional low stringency PCR cycle (eg. 94°C for 15 s, 28°C for 1 min, ramping to 72°C over a period of 3 min, 72°C for 2.5 min), to follow the two high stringency PCR cycles (Figure 2). We tested six different annealing temperatures in these low stringency cycles, using SP1 and various FP primers (Table 1), and found an effect on the final products obtained (see Additional file 2, Figure. S1 and Figure 3 for example of products obtained during cloning of the *FT *ortholog of *Fragaria ananassa*). However, all of the reactions yielded flanking regions and the patterns of amplified fragments after the secondary round of PCR (Figure 3) exhibited strong similarities despite the differences in annealing temperatures of the first PCR step. In many cases, lower annealing temperatures in the low stringency cycles of the first PCR step yielded fewer amplified products (Figure 3). This influence of annealing temperature of the low stringency cycle was confirmed by repeated experiments for the cloning of various genes and T-DNA insertion loci. However, if the low stringency cycle of the first *FPNI-PCR *step was entirely omitted, successful amplification of the target product was still achieved (data not shown).

### Effectiveness and accuracy of FPNI-PCR

Using the *FPNI-PCR *approach, we have successfully isolated 21 complete genomic sequences containing the *FT*, *TFL1 *and *SOC1 *gene orthologs from seven *Rosaceace *species, namely *Spiraea cantoniensis, Pyracantha fortuneana, Photinia serrulata, Fragaria ananassa, Rosa hybrida, Prunus mume *and *Prunus yedoensis*. In addition, we have used *FPNI-PCR *to isolate four *MYB *genes of *Rosa rugosa*, three promoters from the MADS-Box transcription factor family of *Petunia hybrida*, and four T-DNA flanking sequences in transgenic tobacco lines. All of these reactions were conducted using a total of just nine FP primers (FPs), in conjunction with specific primers designed to the conserved/partially known genomic fragments or known T-DNA sequences. Sequences have been deposited in the Genbank library, as shown in Table 3.

**Table 3:** Submitted genomic DNA sequences (genes) in varied species and T-DNA insertion flanking sequences in transgenic tobacco using *FPNI-PCR*.

Code	Accession number	Size (bp)	Anotation
*1*	JF806621	2025	*Photinia serrulata PsFT *gene for flowering locus T protein, complete cds.
*2*	JF806622	2286	*Pyracantha fortuneana PfFT *gene for flowering locus T protein, complete cds.
*3*	JF806623	1474	*Prunus persica PpFT *gene for flowering locus T protein, complete cds.
*4*	JF806624	1695	*Spiraea salicifolia SsFT *gene for flowering locus T protein, complete cds.
*5*	JF806625	1368	*Prunus mume PmTFL11 *gene for *TFL11-like *protein, complete cds.
*6*	JF806626	935	*Rosa hybrida RhTFL11 *gene for *TFL11-like *protein, complete cds.
*7*	JF806627	1794	*Prunus yedoensis PyTFL11 *gene for flowering locus T protein, complete cds.
*8*	JF806628	2230	*Photinia serrulata PsTFL11 *gene for *TFL11-like *protein, complete cds.
*9*	JF806629	1680	*Spiraea cantoniensis ScTFL11 *gene for *TFL11-like *protein, complete cds.
*10*	JF806630	2385	*Pyracantha fortuneana PfTFL11 *gene for *TFL11-like *protein, complete cds.
*11*	JF806631	1252	*Fragaria vesca PmTFL11 *gene for *TFL11-like *protein, complete cds.
*12*	JF806632	4679	*Prunus mume PmSOC1 *gene for *SOC1-like *protein, complete cds.
*13*	JF806633	8200	*Rosa hybrida RhSOC*1 gene for *SOC1-like *protein, complete cds.
*14*	JF806634	5335	*Fragaria vesca FvSOC1 *gene for *SOC1-like *protein, complete cds.
*15*	JF806635	5770	*Photinia serrulata PsSOC1 *gene for *SOC1-like *protein, complete cds.
*16*	JF806636	6053	*Prunus yedoensis PySOC1 *gene for *SOC1-like *protein, complete cds.
*17*	JF806637	4850	*Spiraea cantoniensis SvSOC1 *gene for *SOC1-like *protein, complete cds.
*18*	JF806638	240	Partial T-DNA flanking sequence in the transgenic tobacco plant.
*19*	JF806639	949	Partial T-DNA flanking sequence in the transgenic tobacco plant.
*20*	FR729040	3036	*Prunus mume PmFT *gene for flowering locus T protein, complete cds.
*21*	FR729041	2307	*Rosa chinensis RcFT *gene for flowering locus T protein, complete cds.
*22*	FR729042	2522	*Fragaria vesca FvFT *gene for flowering locus T protein, complete cds.

**Figure 4:**
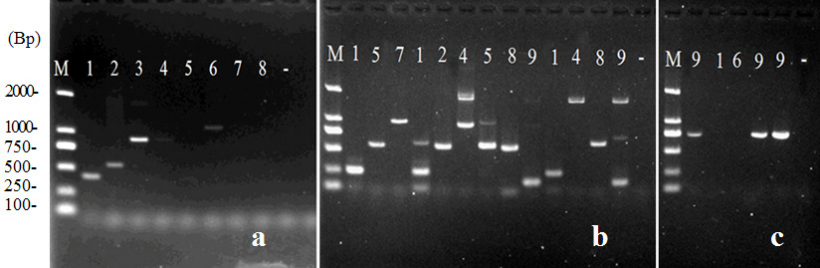
**Amplified products after the tertiary round of PCR in genomic walking and T-DNA flanking sequence cloning using *FPNI-PCR***. (a) Cloning of the *FT-Like *gene of *Rosa rugosa *using 3 gene-specific and 1-8 FP primers. (b) T-DNA flanking sequence cloning in three transgenic tobacco individual plants using forward primers corresponding to the known T-DNA border; various fragment sizes were obtained from the use of different FP primers (1-9) in the three tested tobacco plants. (c) T-DNA flanking sequence cloning using backward primers. Only target specific fragments were amplified by using an appropriate annealing temperature during the low stringency cycles of the first PCR step (similar T-DNA sequences were present in each of the tested transgenic tobacco plants). Note: M: molecular marker; number: 1-9 FP primers; -: control.

Figure 4 shows examples of the products amplified following the tertiary round of PCR in the *FPNI-PCR *method. Figure 4 shows the products relating to cloning of the *FT-Like *gene of *Rosa rugosa*. Figure 4b and 3c show the products arising during the cloning of T-DNA flanking sequences using forward and reverse sequence-specific primers, respectively, in three different transgenic tobacco plants and using various FPs. In addition, based on known genomic sequence fragments, we used *FPNI-PCR *to amplify the various lengths of products from the expected flanking regions of 3 different members of the *wuschel *gene family of *Arabidopsis *(Additional file 2, Figure. S2a), and sequence from the flanking regions of *Osft*, *Osmads1 *and *Ostuba1 *genes of rice (Additional file 2, Figure. S2b). Even for the small DNA molecular size of the pCAMBIA2300 vector, the flanking DNA fragment was amplified successfully using *FPNI-PCR *(data not shown).

**Figure 5:**
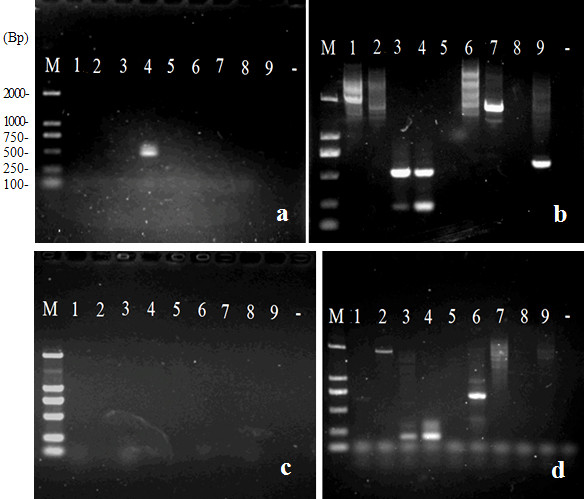
**Comparison of PCR amplification products generated in typical genomic walking experiments using *TAIL-PCR *and *FPNI-PCR *methods, respectively**. (A) Products generated by 3 gene-specific primers and 1-9 arbitrary degenerate primers in *FT *ortholog cloning of *Pyracantha fortuneana *using *TAIL-PCR; *a 510 bp fragment was obtained using the fourth AD primer, only. (B) Products generated by the same 3 gene-specific and 1-9 FP primers in *FT *ortholog cloning of *Pyracantha fortuneana *using *FPNI-PCR*; four specific fragments were amplified by *FPNI-PCR *and the longest fragment was ca. 1.7 kp. (C) Products generated by 3 gene-specific and 1-9 arbitrary degenerate (AD) primers in *SOC1 *ortholog cloning of *Pyracantha fortuneana *using *TAIL-PCR*. (D) Products generated by 3 gene-specific and 1-9 FP primers in *SOC1 *ortholog cloning of *Pyracantha fortuneana *using *FPNI-PCR*. Note: M: molecular marker; number: 1-9 arbitrary degenerate primers (FPs); -: control.

Comparative experiments using the same specific primers showed that, in most cases (data not shown), longer products could be obtained by *FPNI-PCR *than by *TAIL-PCR *(Figure 5a and 5b). Furthermore, *FPNI-PCR *was found to be more reliable as a method of amplifying target genes and unknown flanking sequences. Thus, in a comparative test using several appropriate AD primers, *TAIL-PCR *frequently failed to amplify the predicted DNA fragment. Figure 5a shows the results of *TAIL-PCR *in the cloning of the *FT *ortholog from *Pyracantha fortuneana *using 3 gene-specific primers and the FP1-9 arbitrary degenerate primers. In this experiment, a 510 bp fragment was obtained from *TAIL-PCR *with just one (i.e. the fourth) AD primer. By contrast, Figure 5b shows the products generated by *FPNI-PCR *using the same primers for the same genome walking experiment. Here, four of the *FPNI-PCR *reactions amplified specific fragments, and the longest fragment was ca. 1.7 kp. Genomic walking for the *SOC1-like *gene in *Pyracantha fortuneana *provided another situation in which we encountered "off-target" results using *TAIL-PCR*, whereas, using the *FPNI-PCR *method we obtained two specific fragments of 2068 bp and 683 bp (Figure 5c and 5d). Sequence analysis of 102 amplified DNA fragments resulting from the *FPNI-PCR *technique showed that they corresponded with 100% accuracy to the target products.

### Efficiency of FPNI-PCR

*FPNI-PCR *facilitates the rapid identification of target genomic sequences by permitting the use of short-cuts for a number of the steps between tissue isolation and target product sequencing, thereby reducing the input of time and effort compared to other current methods of flanking sequence cloning. For example, *FPNI-PCR *can be employed to generate target genomic products when cell lysates are used to supply the DNA templates (Figure 4). For this procedure, we extracted cell lysates from the young leaves of transgenic tobacco, *petunia hybrida *and *Rosa hybrida *using the rapid NaOH extraction method. We found no difference in the success of target product amplification from these cell lysates compared to that from the higher quality DNA templates as purified by the CTAB method. In addition, we found no differences between the results of direct sequencing of the *FPNI-PCR *products compared to indirect sequencing of the cloned products within the PMD18-T vector. *FPNI-PCR *has the advantage that, after the first step (12-15 cycles), we can proceed to the second step directly, without requiring the prior dilution of the products from the first PCR step. When using such practices, we commonly achieved > 85% positive cloning of target products following the secondary PCR reaction. Thus, we were able to amplify target products using just 42-45 PCR cycles, and this was completed in less than 3 hours from the start of tissue extraction. When proceeding through the third PCR and final sequencing steps, *FPNI-PCR *was found to provide 100% positive results in our working tests of genomic cloning. In total, the procedure was completed in just 54-57 cycles, taking less than 4 hours.

## Discussion

### FPNI-PCR involves a novel strategy of amplification

The *FPNI-PCR *strategy is based on the following technical points. (1) The arbitrary degenerate oligonucleotides forming the 3' section of the FP primers (FPs) have been designed with the intention of maximizing the level of similarity with common genomic oligo sequences throughout the various taxa. (2) During the first PCR step, the 3'-ends of the oligos forming the arbitrary degenerate portions of the FPs (which also include several fixed bases at the 3'-end) need to pair effectively with selected sites of high similarity in the single-stranded DNA template. A lower annealing temperature may be used to increase mismatch sites and, thereby, enlarge the universality of the designed fusion primers. Between 1 and 6 cycles in the first PCR step are used to ensure that the desired target products are present within the amplified mix. (3) In the secondary and tertiary PCR steps, the target DNA should be exponentially amplified via high stringency cycles, while non-target products are not amplified by the gene-specific and FP-specific primer pairs. Off-target amplification may also be suppressed by the stem-loop structure of nonspecific DNA products. The use of high stringency PCR cycles encourages the efficient and specific amplification of the target products, while off-target PCR products are progressively diluted. This aspect of the *FPNI-PCR *strategy represents a key difference from the *TAIL-PCR *method developed by Liu and Whittier [1]. *TAIL-PCR *employs multiple low stringency annealing cycles and, thus, nonspecific products are readily amplified. In *TAIL-PCR*, enrichment of the target products relies upon the difference in amplification velocity between the target and non-target products. Thus, the *TAIL-PCR *protocol involves two rounds of laborious DNA dilutions and numerous (approx. 110) cycles of PCR [1]. An improved method, named *hiTAIL-PCR*, similarly involves two rounds of laborious DNA dilutions and multiple low and high stringency PCR cycles, thereby still providing opportunities for nonspecific product amplification [3]. In addition, *hiTAIL-PCR *is uneconomical in terms of primer design, with the secondary gene-specific primers requiring the addition of > 20 nucleotides [3].

### FPNI-PCR represents a very powerful tool in genomic walking and flanking sequence cloning

*TAIL-PCR *has been widely used for genome walking and flanking sequence cloning in plants and other organisms. Commonly, however, specific products appear in the second round of PCR only to disappear during the third round of PCR [17]. Liu and Chen reported that just 60% of reactions yielded specific products [3]. Our own tests of the *TAIL-PCR *method found that, on some occasions, no product was observed even after three rounds of PCR. Generally, smaller and fewer products were generated by *TAIL-PCR *as compared to the corresponding reactions using *FPNI-PCR *(Figure 5).

For inverse and ligation-mediated PCR-based genome-walking methods, successful amplification of target products relies on the restriction fragmentation of genomic DNA. When employing such techniques, we also encountered some "off-target" situations, possibly due to the location of the available restriction sites relative to the locus-specific primer [6]. By contrast, repeated tests of the *FPNI-PCR *method using the nine given FP primers revealed no "off-target" cases, and more than 80% of *FPNI-PCR *reactions yielded DNA fragments larger than 1.0 kb. Thus, our results indicate that *FPNI-PCR *is a more powerful technique for genomic walking and flanking sequence cloning studies. In addition, the *FPNI-PCR *method makes it feasible to amplify large sections of specific DNA fragments. This can be achieved through the control of annealing temperatures during the low stringency cycles of the first PCR step. The control of this parameter can also be used to avoid the cloning of "off target" flanking sequences.

### FPNI-PCR is a simple and rapid method of genomic walking and flanking sequence cloning

Methods involving inverse PCR and ligation-mediated PCR techniques suffer from the requirement for complicated manipulations, such as restriction cleavage, ligation, or tailing before PCR amplification [6,7,9]. Consequently, the use of such methods to successfully amplify and clone the target product usually requires a time-scale exceeding one working day. Similarly, the successful application of *TAIL-PCR *or *hiTAIL-PCR *methods is also time-consuming, with the time taken to perform the necessary number of PCR cycles (approx. 110) requiring a full working day (and this is even before the time taken to perform the various dilution steps is considered) [1,3]. Using *FPNI-PCR*, a total about 42-45 cycles (requiring less than two hours) can be adequate to produce a good rate of success in target product amplification and, in our hands, a total of 54-57 cycles (completed in less than 4 hours) ensured a successful result in 100% of cases. Both *TAIL-PCR *and *FPNI-PCR *methods demand only extremely modest levels of quantity and purity of the template DNA, thus, they can work well with both cell lysates and crude DNA extracts. Using *FPNI-PCR*, however, we were able to choose a wider range of annealing temperatures during the low stringency cycles of the first PCR step, thereby extending the scope of target product that could be successfully amplified. The currently widely used Genome Walker Kit from Clontech employs restriction enzyme cleavage of DNA and adaptor-ligated genomic DNA construction, and also involves a nested PCR strategy. The procedures of integrated restriction cleavage, ligation and tailing make the Genome Walker Kit a substantially more complicated and time-consuming process than the first PCR step of *FPNI-PCR*. Thus, we believe that in comparison to the other reported methods of genomic walking and flanking sequence cloning [[3,15,18,19], and references as above], *FPNI-PCR *is technically less demanding and is more time-efficient.

### FPNI-PCR is highly effective and accurate

We conducted 60 rounds of *FPNI-PCR *reactions using the nine given FP primers, and in each case we successfully amplified products and experienced no "off-target" results. This contrasts to the experiences when employing ligation-dependent PCR methods, when it is often a problem to find available restriction sites and obtain ligation products [16]. In addition, sequence analysis of 102 amplification products from *FPNI-PCR *indicated that the accuracy of positive cloning in our experiments was 100%. By comparison, 60-80% of reactions yielded specific products according to the original *TAIL-PCR *technical report [1], and a success rate of 93% was recorded for the improved *hiTAIL-PCR *procedure [3]. Thus, we conclude that *FPNI-PCR *shows a substantially higher level of effectiveness than the established *TAIL-PCR *procedures. We suggest that the high rate of positive amplification results, at least partially, from the high stringency PCR cycles in the second and third PCR stages of the *FPNI-PCR *protocol.

## Conclusion

To date, *TAIL-PCR *has been widely used for genome walking and flanking sequence cloning in molecular biology research, but it is still laborious and time-consuming. Recently, several alternative methods have been introduced [2,6,17], including the Genome Walker Kit from Clontech, but these methods still involve the use of restriction enzyme-mediated digestion, Phi29 DNA polymerase-mediated amplification, and a series of template DNA purification steps. Here, we report a simple and effective PCR method namely, fusion primer and nested integrated PCR (*FPNI-PCR*). This method is based on the use of arbitrary degenerate nucleotides reflecting natural restriction sites or site-dependent nucleotides in the genomic DNA to design universal primers. These FP primers are used to conduct the first round of PCR, which is followed by two to three rounds of nested PCR. We undertook a large number of experiments using *FPNI-PCR *in order to robustly demonstrate that this novel method has the capability to be more powerful, effective and accurate than the established *TAIL-PCR *method. Furthermore, the *FPNI-PCR *method is completed in a shorter time-scale than other widely-used genome-walking techniques, and is significantly less technically complex to perform.

## Methods

### Genomic DNA isolation

Genomic DNA of seven *Rosaceace *species, namely, *Pyracantha fortuneana, Prunus mume, Photinia serrulata L., Prunus persica, Spiraea cantoniensis L., Rosachinensis *and *Fragaria × ananassa*, was extracted from young leaves according to the procedure of Yang et al [20]. In addition, genomic DNA of tobacco, *Petunia hybrida*, *Arabidopsis *and rice was isolated from young leaves according to the NaOH fast extraction method, as reported previously [21].

### Oligonucleotide primers

All sequence specific primers were designed using Primer5 software (see Additional files 1, Table S4), and annealing (Tm) temperatures ranged from 60°C to 72°C. For the design of the arbitrary degenerate primers (ADs), AD sequences were either collected from the existing literature or were designed by us (Additional files 1, Table S1). The AD oligos were fused to the 3' end of an adaptor oligo which supplied a known sequence of 31 fixed (i.e. non-degenerate) nucleotides, thereby forming the fusion primers (FPs). The simplest form of these FPs is termed type I primers (Table 1). Type II primers are designed to include a hairpin structure within the 5' end of the single-stranded adaptor regions (Additional files 1, Table S2). Type III primers are arbitrary degenerative primers that contain just 4-6 fixed nucleotides at the 3' end (Additional files 1, Table S3). Figure 1a illustrates the schematic relationship between the sequence-specific and arbitrary degenerate primers with the target genomic sequence or flanking sequence of a T-DNA insertion. All primers used in this study were synthesized by Invitrogen Co. Limit, and are summarized in Table 1 and Additional files 1, Tables S1-S5. The annealing (Tm) temperatures of the arbitrary degenerate primers (ADP) used in this study ranged between 36°C and 48°C. Tm values were estimated by using the formula: Tm = 81.5 + 16.6 × Log_10_[Na+] + 0.41 (%GC) - 600/total number of nucleotides.

### PCR conditions, procedures and gel analysis methods

All *FPNI-PCR *procedures were arranged in three stages. The first stage involved a PCR step which was performed in a total volume of 20 μL, and contained 1-2 μL of the DNA products (i.e. 2 μL DNA extraction solution from the NaOH fast extraction method), 0.2 μM gene-specific primers (SP1), 1.0 μM fusion arbitrary degenerate primers and 1 U rTaq (Takara). Between 9-18 PCR cycles were performed using annealing temperatures ranging between 28°C and 52°C (full details of the conditions used are given in Table 2). In the second and third PCR stages, gene-specific primers (SP2 and SP3) and adaptor-specific primers (FSP1 and FSP2) were used in place of the SP1 and fusion arbitrary degenerate primers, respectively; the schematic arrangement of these primer pairs with respect to the target genomic DNA is illustrated in Figure 2a. Due to the exact primer-template match for the primer pairs used in the second and third PCR steps, high stringency cycles were employed in these two latter stages (details shown in Table 2). For the second PCR step, a 1.0 μL aliquot of the first PCR product pool was used as the template. Following this second round of selective amplification, the secondary PCR products were diluted 50-fold with MQ water and 1 μL of the dilution was added as template to 20 μL volumes of the tertiary PCR mixtures. For visualisation of the products amplified following the second or third *FPNI-PCR *steps, a 5-8 μL aliquot was separated by electrophoresis in a 1.5% agarose TAE gel, then stained with ethidium bromide and observed under a UV illumination system.

### DNA sequencing

For direct sequencing, the remaining 12-15 μL of PCR products were purified through a Sephadex G-50 column and subjected to sequencing procedures using SP3 primers designed to known sections of the target sequence, or FSP2 primers designed according to the original FP primers. For indirect sequencing, bands corresponding to the largest products were excised from the agarose gels, purified and cloned into a pMD18-T TA cloning vector (TaKaRa), and then sequenced with M13 forward or reverse primers.

## Authors' contributions

ZW, SY and JL performed the experiments. ZW drafted the manuscript. BZ and MB finalized the paper. GN conceived and developed the *FPNI-PCR *procedure and supervised the project. All authors read and approved the final manuscript.

## Supplementary Material

Additional file 1 **Tables for listing different used primers. Table S1**. AD primer (arbitrary degenerate primers) and universal primers used in this study. **Table S2**. FP primers (exhibiting restriction site) and the corresponding universal primers used in *FPNI-PCR*. **Table S3**. FP primers (exhibiting hair pin structure) and the corresponding universal primers used in *FPNI-PCR*. **Table S4**. Gene specific primers used to generate the electrophoresis patterns presented in this paper. **Table S5: **Gene specific primers used to generate the electrophoresis patterns presented when conducting genomic walking in *Arabidopsis *and *rice*. Click here for file

Additional file 2 **Supporting figures employed in main text. Figure S1**. Illustration of the effect of annealing temperature during the low stringency PCR cycles in the primary PCR step in *FT *ortholog cloning of *Fragaria ananassa *using *FPNI-PCR *(amplified products after the secondary round of PCR). M: molecular marker; number in the lanes: 1-9 FP primer; number in the bottom right corner: annealing temperature. -: control. **Figure S2**. Amplified products after the tertiary round of PCR in genomic walking and T-DNA flanking sequence cloning using *FPNI-PCR *for the 3 genes of *wuschel *family from *Arabidopsis *(a), and *Osft, Osmads1 *and *Ostuba1 *genes from rice (b) (FP primer indicated by number in photos). Click here for file
