# Supplementary material for: Fusion primer and nested integrated PCR (FPNI-PCR): a new high-efficiency strategy for rapid chromosome walking or flanking sequence cloning
Source: BMC Biotechnol. 2011 Nov 17;11:109. doi: 10.1186/1472-6750-11-109 (PMC3239319; doi:10.1186/1472-6750-11-109)
Supplement: Additional file 1 — Tables for listing different used primers. Table S1. AD primer (arbitrary degenerate primers) and universal primers used in this study. Table S2. FP primers (exhibiting restriction site) and the corresponding universal primers used in FPNI-PCR. Table S3. FP primers (exhibiting hair pin structure) and the corresponding universal primers used in FPNI-PCR. Table S4. Gene specific primers used to generate the electrophoresis patterns presented in this paper. Table S5: Gene specific primers used to generate the electrophoresis patterns presented when conducting genomic walking in Arabidopsis and rice. [file 1472-6750-11-109-S1.PDF]

Additional files

Additional file1:

Title: Tables for listing different used primers.

Description:

Table S1. AD primer (arbitrary degenerate primers) and universal primers used in this study.

Table S2. FP primers (exhibiting hair pin) and the correspondence universal primers used in *FPNI-PCR*.

Table S3. FP primers (exhibiting restriction site) and the correspondence universal primers used in *FPNI-PCR*.

Table S4. Gene specific primers used to generate the electrophoresis pattern presented in this paper.

Table S5: Gene specific primers used to generate the electrophoresis pattern presented when using genes' genomic walking in *Arabidopsis* and *Rice*.

Table S1: AD primer (arbitrary degenerate primers) and universual primers used in this study.

Name	*Primer sequence*	*Primer use*
ad1:	NTCGA STWTS GWGTT	1,2,3rd PCR primer
ad2:	NGTCG ASWGA NAWGAA	1,2,3rd PCR primer
ad3:	WGTGN AGWAN CANAGA	1,2,3rd PCR primer
ad4:	AGWGN AGWAN CAWAGG	1,2,3rd PCR primer
ad5:	NGTAW AASGT NTSCA A	1,2,3rd PCR primer
ad6:	NGACG ASWGA NAWGAC	1,2,3rd PCR primer
ad7:	NGACG ASWGA NAWGAA	1,2,3rd PCR primer
ad8:	GTNCG ASWCA NAWGTT	1,2,3rd PCR primer
ad9:	NCAGC TWSCT NTSCTT	1,2,3rd PCR primer

Table S2: FP primers (exhibiting hair pin) and the correspondence universal primers used in *FPNI-PCR*.

Name	*Primer sequence*	*Primer use*
Fpad1-1:	CGTGCGTATATCGACTCACTATAGGGCACGCGTGGT NTCGA STWTS GWGTT	1st PCR primer
Fpad1-7:	CGTGCGTATATCGACTCACTATAGGGCACGCGTGGT GTNCG ASWCA NAWGTT	1st PCR primer
Fpad1F1:	GCGTATATCGACTCACTATAGGGC	2nd PCR primer
Fpad1F2:	CACTATAGGGCACGCGTGGT	3rd PCR primer
Fpad2-1:	CGTGCCTATATCGACTCACTATGAGGCACGCGTGGT NTCGA STWTS GWGTT	1st PCR primer
Fpad2-7:	CGTGCCTATATCGACTCACTATGAGGCACGCGTGGT GTNCG ASWCA NAWGTT	1st PCR primer
Fpad2F1:	GCCTATATCGACTCACTATGAGGC	2nd PCR primer
Fpad2F2:	CACTATGAGGCACGCGTGGT	3rd PCR primer
Fpad3-1:	CGTGCCTATATCGACTCACTGATAGGCACGCGTGGT NTCGA STWTS GWGTT	1st PCR primer
Fpad3-7:	CGTGCCTATATCGACTCACTGATAGGCACGCGTGGT GTNCG ASWCA NAWGTT	1st PCR primer
Fpad3F1:	GCCTATATCGACTCACTGATAGGC	2nd PCR primer
Fpad3F2:	CACTGATAGGCACGCGTGGT	3rd PCR primer
Fpad4-1:	CGTACTAAGACTCACTACAGGGTACGCGTGGT NTCGA STWTS GWGTT	1st PCR primer
Fpad4-7:	CGTACTAAGACTCACTACAGGGTACGCGTGGT GTNCG ASWCA NAWGTT	1st PCR primer
Fpad5-1:	CGCGTACTAAGACTCACTACAGGGTACGCGTGGT NTCGA STWTS GWGTT	1st PCR primer
Fpad5-7:	CGCGTACTAAGACTCACTACAGGGTACGCGTGGT GTNCG ASWCA NAWGTT	1st PCR primer
Fpad4&5F1:	CGTACTAAGACTCACTACAGGGT	2nd PCR primer
Fpad4&5F2:	CACTACAGGGTACGCGTGGT	3rd PCR primer

Table S3: FP primers (exhibiting restriction site) and the correspondence universal primers used in *FPNI-PCR*.

Name	Primer sequence	Primer use
Hind-dep:	GTAATACGACTCACTATAGGGCACGCGTGG NNNNN AAGCTT	1st PCR primer
EcoR1-dep:	GTAATACGACTCACTATAGGGCACGCGTGG NNNNN GAATTC	1st PCR primer
Pst1-dep:	GTAATACGACTCACTATAGGGCACGCGTGG NNNNN CTGCAG	1st PCR primer
Site1-dep:	GTAATACGACTCACTATAGGGCACGCGTGG NNNNN GACTC	1st PCR primer
Site2-dep:	GTAATACGACTCACTATAGGGCACGCGTGG NNNNN GATC	1st PCR primer
FSP1:	GTAATACGACTCACTATAGGGC	2nd PCR primer
FSP2:	ACTATAGGGCACGCGTGGT	3rd PCR primer

Table S4: Gene specific primers used to generate the electrophoresis pattern presented in this paper.

Experements	*Name*	*Primer sequence*	*Primer use*
T-DNA flanking sequence clone	***NptF1:***	ATTGCTGAAGAGCTTGGCGGCGAAT	1st PCR primer
	NptF2:	GACCGCTTCCTCGTGCTTTACGGTAT	2nd PCR primer
	NptF3:	CTATCGCCTTCTTGACGAGTTCTTCTGA	3rd PCR primer
	NptR1:	GGCATCAGAGCAGCCGATTGTCTGTTGT	1st PCR primer
	NptR2:	GTCATAGCCGAATAGCCTCTCCACCCA	2nd PCR primer
	NptR3:	CCTGCGTGCAATCCATCTTGTTCAATCA	3rd PCR primer
Genomic walking	***PfFtsp1***	ATTCCTTTAGGTTGGGGTCACTTG	1st PCR primer
	PfFtsp2	GGATGGATGGAAGATGGAAACTTACC	2nd PCR primer
	PfFtsp3	TCCTTAGTACCGTAGGTCACCCTCAG	3rd PCR primer
	FrFtsp1	CCGCCGTTGTTGCCGGAATATCAGT	1st PCR primer
	FrFtsp2	TGGACTGGGCGCATCAGGATCTACC	2nd PCR primer
	FrFtsp3	CCCTGTTATTACTGCAAGTCATCCTC	3rd PCR primer
	PfSoc1sp1	CGGCTTAGTCCATATTAATCTCCCCTGTC	1st PCR primer
	PfSoc1sp2	GCGAACACCAATAATACATAGGACATCA	2nd PCR primer
	PfSoc1sp3	GGAGTCTAGTGTTAGATTGGACATTGATT	3rd PCR primer
	RrMybsp1	ATGTAGTAGTGTTTTTGTG	1st PCR primer
	RrMybsp2	CGTTGGTGGAGGAATTAGC	2nd PCR primer
	RrMybsp3	TCTGACTTGAGCCCCTACT	3rd PCR primer

Table S5: Gene specific primers used to generate the electrophoresis pattern presented when using genes' genomic walking in *Arabidopsis* and *Rice*.

Crop species	*Name*	*Primer sequence*	*Primer use*
	Atwus13sp1	CCTCCACCATGCTGCTTCCTCTTG	1st PCR primer
	Atwus13sp2	GGAGTCCACCTCTGTCTAGCTGTCA	2nd PCR primer
	Atwus13sp3	GCACATCATCAAGCTAAAAACACCT	3rd PCR primer
	Atwus1sp1	TGCTAAACTCAGAACCTCAACATCCAC	1st PCR primer
Arabidopsis	***Atwus1sp2***	ATAGCCCGAGAACGCCAGAAACGA	2nd PCR primer
	Atwus1sp3	CCTCCAGGTAAGGGACCATAACAG	3rd PCR primer
	Atwus2sp1	TGGCAAACGAAGTAAACGCAGGAAC	1st PCR primer
	Atwus2sp2	TCAATCGCCTCCTCCACAAAACCTC	2nd PCR primer
	Atwus2sp3	GCTGACTTATCCGTATCTAATCTTCG	3rd PCR primer
	Atwus11sp1	CATAGCCCAACCCGCCATAGTCG	1st PCR primer
	Atwus11sp2	CGGACGCAACCACCAACACTT	2nd PCR primer
	Atwus11sp3	GGTCTGGAGGTTGTAGCACTT	3rd PCR primer
	Osft1	TTCGTCCGGATCACTAACCTCAG	1st PCR primer
	Osft1	GGTAGGCACCGATCAGATATGTTAG	2nd PCR primer
Rice	***Osft1***	TCTGTCTGTAGGCTGGTCACCGATA	3rd PCR primer
	Osmads1	GTCCTAGCCTCCTTGTCCGTTTGC	1st PCR primer
	Osmads1	CCTCCCAGCCACTACTACGCTTTTA	2nd PCR primer
	Osmads1	CGAGACCCCAAACTCCAAGCATAC	3rd PCR primer
	Ostuba1	GCGACGTAAAGTTGAGGGACAGAGT	1st PCR primer
	Ostuba1	TCGCAACGACACGGACTTTCTAC	2nd PCR primer
	Ostuba1	CATCCACAGCCAAGCCAACGGT	3rd PCR primer
