# Supplementary material for: Fusion primer and nested integrated PCR (FPNI-PCR): a new high-efficiency strategy for rapid chromosome walking or flanking sequence cloning
Source: BMC Biotechnol. 2011 Nov 17;11:109. doi: 10.1186/1472-6750-11-109 (PMC3239319; doi:10.1186/1472-6750-11-109)
Supplement: Additional file 2 — Supporting figures employed in main text. Figure S1. Illustration of the effect of annealing temperature during the low stringency PCR cycles in the primary PCR step in FT ortholog cloning of Fragaria ananassa using FPNI-PCR (amplified products after the secondary round of PCR). M: molecular marker; number in the lanes: 1-9 FP primer; number in the bottom right corner: annealing temperature. -: control. Figure S2. Amplified products after the tertiary round of PCR in genomic walking and T-DNA flanking sequence cloning using FPNI-PCR for the 3 genes of wuschel family from Arabidopsis (a), and Osft, Osmads1 and Ostuba1 genes from rice (b) (FP primer indicated by number in photos). [file 1472-6750-11-109-S2.PDF]

Additional files

Additional file 2:

Title: Supporting figures employed in main text.

Description:

Figure S1. Illustration of the effect of annealing temperatures in the low stringency PCR cycles in the primary PCR in *FT* ortholog cloning of *Fragaria ananassa* using *FPNI-PCR* (amplified products after the secondary round of PCR). M: molecular marker; number in the lanes: the 1-9 FP primer; number in the bottom right conner: annealing temperatures used. -: control.

Figure S2. Amplified products after the tertiary round of PCR in genomic working and T-DNA flanking sequence in *FPNI-PCR* in the 4 genes of *wuschel* family from *Arabidopsis* (a), and *Osft*, *Osmads1* and *Ostuba1*genes from *Rice* (b) using a certain FP primer indicated by number in photos.

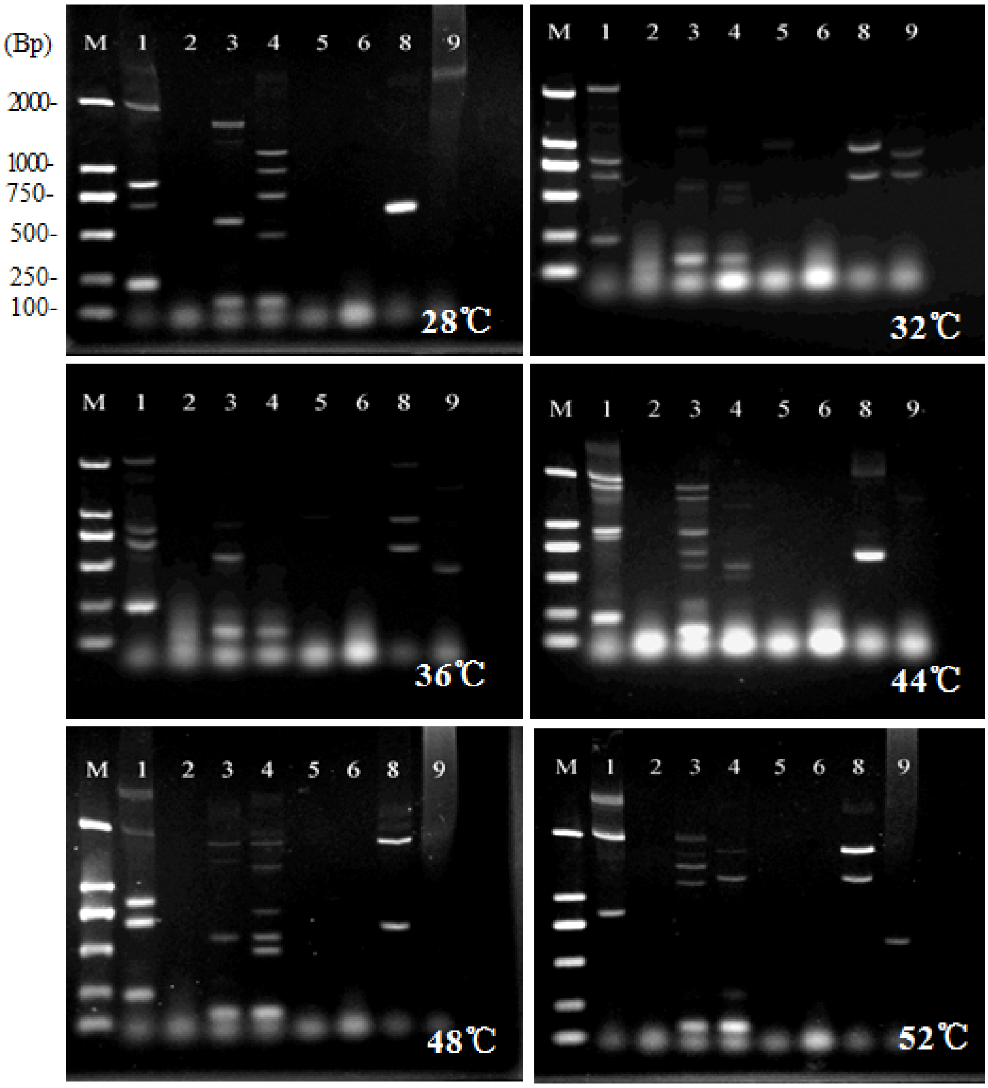

Figure.S1

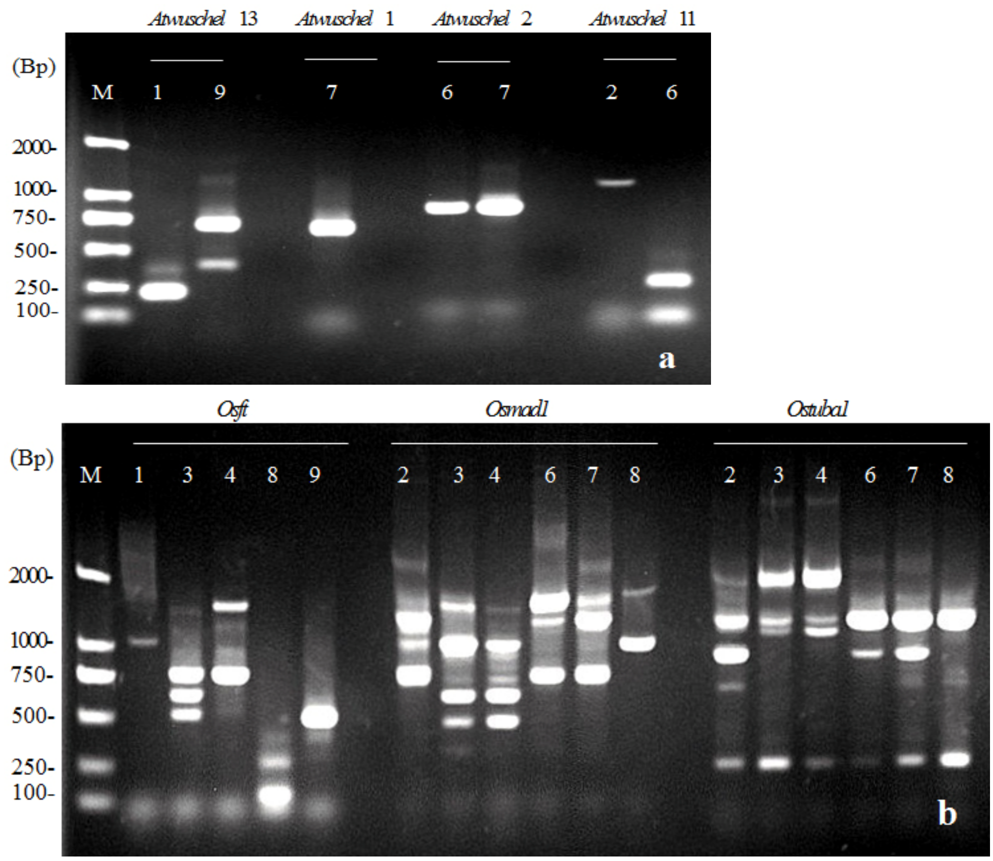

Atwuschel 13
Atwuschel 1
Atwuschel 2
Atwuschel 11
(Bp)
M 1 9 7 6 7 2 6
2000-
1000-
750-
500-
250-
100-
a
Osft
Osmadl
Ostubal
(Bp)
M 1 3 4 8 9 2 3 4 6 7 8 2 3 4 6 7 8
2000-
1000-
750-
500-
250-
100-
b

Figure.S2
